# Design and initial findings of a natural history study of anal HPV and associated lesions among young adult women in Costa Rica

**DOI:** 10.1002/ijc.70082

**Published:** 2025-08-15

**Authors:** Cameron B. Haas, Rebeca Ocampo, Danping Liu, Michael Zúñiga, Diego Guillen, Megan A. Clarke, Loretto J. Carvajal, Allan Hildesheim, John Schussler, Mónica Sierra, Teresa M. Darragh, Joel M. Palefsky, Carolina Porras, for the Costa Rica HPV Vaccine Trial (CVT) Group, Bernal Cortés, Paula González, Rolando Herrero, Silvia E. Jiménez, Carolina Porras, Ana Cecilia Rodríguez, Allan Hildesheim, Aimée R. Kreimer, Douglas R. Lowy, Mark Schiffman, John T. Schiller, Mark Sherman, Ligia A. Pinto, Troy J. Kemp, Leidos Biomedical Research, Frederick National Laboratory, Cancer Research, Mary K. Sidawy, Wim Quint, Joel M. Palefsky, Mark H. Stoler, Aimée R. Kreimer, Rolando Herrero

**Affiliations:** ^1^ Division of Cancer Epidemiology and Genetics National Cancer Institute Bethesda Maryland USA; ^2^ Agencia Costarricense de Investigaciones Biomédicas‐Fundación INCIENSA (ACIB‐FUNIN) San José Costa Rica; ^3^ Translational Oncology Shared Resource Cedars Sinai Medical Center Los Angeles California USA; ^4^ Information Management Services (IMS) Rockville Maryland USA; ^5^ University of California‐San Francisco San Francisco California USA

**Keywords:** anal cancer screening, anal dysplasia, anal HPV

## Abstract

Anal cancer is caused by HPV, and its incidence has increased globally over the last five decades. We conducted an anal HPV natural history study nested in the Costa Rica HPV Vaccine Trial (CVT) to quantitatively describe the natural history and clinical outcomes of anal HPV in HIV‐negative women. CVT was a randomized clinical trial of the bivalent HPV vaccine, and an anal substudy included women selected based on a priori criteria to enrich for anal disease. For women in the anal substudy, anal samples were collected for cytology and determination of HPV16/18/45 for up to four annual visits. Women were referred for high‐resolution anoscopy (HRA) based on abnormal anal cytology or anal HPV16/18/45 positivity. We describe the analytic cohort for this study according to inclusion criteria and factors associated with referral criteria for HRA during study follow‐up. 9,416 women, 1,178 were included in the anal substudy (age‐range = 23–35‐years): 93 had prevalent anal HPV16, 236 had cervical HPV16, 364 had an anal high‐risk HPV infection other than 16, 295 had anal low‐risk HPV, and 227 were included as anal HPV‐negative controls; 352 women were included because of a CIN3+ in study follow‐up. From 3,033 study visits over a median of 70 months follow‐up, 71 (6%) women were referred to HRA. This anal study nested within the CVT infrastructure and enriched for anal disease provides a unique setting to address research questions related to the natural history of anal HPV infections and associated lesions in women without HIV.

## What's New?

Infection with human papillomavirus (HPV) is the primary cause of anal cancer, for which the majority of cases occur in women. Anal cancer screening guidelines have limited evidence to inform effective strategies for women with HIV. We designed a study that was nested within the Costa Rica HPV Vaccine Trial to understand the natural history of anal HPV infection and referred high‐risk women to receive high‐resolution anoscopy (HRA). From 10,302 women in CVT, we followed 1,178 women over 3,033 annual visits and referred 71 to HRA. These findings will inform our understanding of the natural history of anal HPV infection and inform guidelines for anal cancer screening programs.

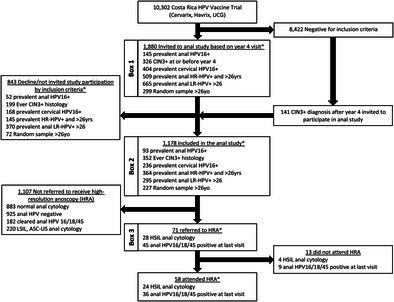

**Abbreviations**
AIN: Anal intraepithelial neoplasia
ANCHOR: Anal Cancer/HSIL Outcomes Research
CI: Confidence interval
CIN: Cervical intraepithelial neoplasia
CVT: Costa Rica HPV Vaccine Trial
DEIA: DNA enzyme immunoassay detection of amplimers
HPV: Human papillomavirus
HR: High‐risk
HRA: High‐resolution anoscopy
HSIL: High‐grade squamous intraepithelial lesion
LiPA: Line probe assay
LR: Low‐risk
LTFU: Long‐term follow‐up
MSM: Men who have sex with men
PCR: Polymerase chain reaction
PR: Prevalence ratio
PWH: People with HIV
UCG: Unvaccinated control group

## 1 BACKGROUND

The incidence of anal cancer has been rising globally by 3%–6% annually in most countries.^1^ An estimated 91% of anal squamous cell carcinomas are caused by carcinogenic human papillomavirus (HPV) infection, particularly HPV16.^2^ While men who have sex with men (MSM) with HIV have the highest reported incidence rate of anal cancer, they contribute only a modest fraction (~9%) to the burden of anal cancers each year despite the elevated risk.^3^ In the general population, females have a higher incidence of anal cancer than males and represent approximately two‐thirds of cases, and approximately 1% are among women with HIV.^3^, ^4^ In Costa Rica, anal cancer incidence is 4‐times greater in females than in males, which is similar to what has been observed in many countries with a high Human Development Index.^5^

Among HIV‐negative women, estimates of anal HPV prevalence vary widely, and few cohort studies have longitudinal data for estimating incidence and clearance of anal HPV.^6^ A systematic review showed that most prior studies recruited women from colposcopy clinics, biasing anal HPV estimates to represent a population with higher rates of abnormal cervical cytology and cervical HPV.^6^ In a study of sexually active women (mean age 39) over 1.2 years of average follow‐up, the incident anal carcinogenic HPV infection rate was 19.5/1000 person‐months.^7^ However, longer follow‐up is necessary to detect incident anal HPV infection and subsequent clearance of new infections, as prevalent infections are likely biased toward infections with longer persistence and thus more strongly related to disease. Accurate estimates of incidence, clearance, and duration in representative samples of women from the general population are essential to understanding the natural history of anal HPV infection and disease risk.

Anal high‐grade squamous intraepithelial lesion (HSIL; which includes severe dysplasia or anal intraepithelial neoplasia [AIN3] and p16‐positive AIN2) is a precursor to squamous cell carcinoma. The gold standard approach for detecting anal HSIL is high‐resolution anoscopy (HRA), with HRA‐guided biopsy.^8^ In some settings, anal cytology is used as an initial screening test to identify patients who are at risk for anal precancer and require HRA referral.^9^ Results from the Anal Cancer/HSIL Outcomes Research (ANCHOR) trial showed that, among people with HIV (PWH), detection and treatment of anal HSIL can reduce the 4‐year cumulative incidence of anal cancer by 57%.^10^ This raises the question of whether the documented benefits of anal cancer screening in PWH may extend to some HIV‐negative women, especially subgroups with elevated risk such as women with genital precancer (i.e., cervical, vulvar, or vaginal).^11^ Describing the natural history of anal HPV and the detection of anal HSIL in HIV‐negative women is necessary to inform considerations of the utility of anal cancer screening in immunocompetent women.

The Costa Rica HPV Vaccine Trial (CVT) was a large, community‐based clinical trial that evaluated the efficacy of HPV vaccination against cervical HPV infections and associated lesions; including the investigation of anal HPV vaccine efficacy.^12^ The expansion to anal endpoints provided the impetus for the anal natural history study nested within CVT, whose study design and population are described in this manuscript. In addition, we describe the referral patterns to HRA based on abnormal anal cytology and anal HPV infection.

## 2 MATERIALS AND METHODS

### 2.1 Study design

The CVT was a double‐blind, controlled, randomized, phase III study of the efficacy of the ASO4‐adjuvanted HPV‐16/18 vaccine for prevention of HPV infection.^13^ During 2004–2005, a community‐based sample of 7466 women (18–25 years) was recruited and randomized to receive three doses of the bivalent HPV vaccine (Cervarix®, GlaxoSmithKline Biologicals; Rixensart, Belgium) or a control hepatitis A virus vaccine (Havrix, GlaxoSmithKline Biologicals; Rixensart, Belgium), and were followed for 4 years.^13^ At the year‐four study visit, in 2009 to 2010, collection of anal swab specimens was introduced to determine anal HPV status.^12^ At the conclusion of the blinded phase of the trial, the original control arm was provided the HPV vaccine and exited from the study. A new unvaccinated group—referred to as the unvaccinated control group (UCG; *N* = 2836)—was recruited contemporaneously at the study visit where vaccination of the original control arm was done, to measure attack rates of HPV infections and disease compared to the HPV‐vaccinated group in a long‐term follow‐up study (LTFU).^14^

**FIGURE 1:**
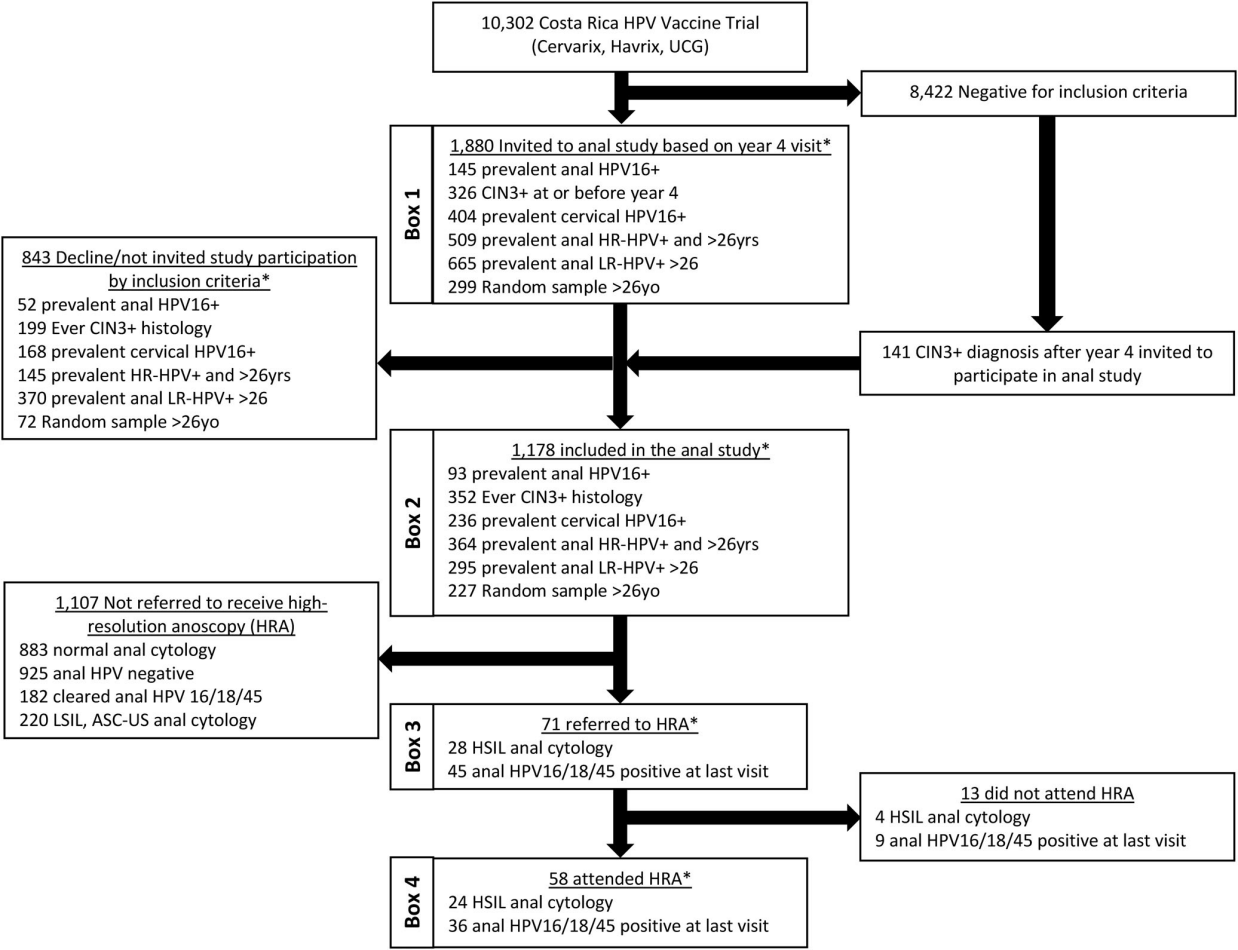
Study population, with inclusion and exclusion criteria and referral to high‐resolution anoscopy (HRA). CIN3+, Cervical intraepithelial neoplasia grade; HPV, Human papillomavirus; HR, Non‐16 carcinogenic HPV; LR, Low‐risk/non‐carcinogenic; UCG, Unvaccinated control group. *Inclusion criteria not.

The anal substudy was embedded in the larger investigation described above. For efficiency, only a subset of participants from the main study were invited to participate in the anal substudy based on a priori risk factors for anal disease. Six subgroups were included to cover a broad range of anal cancer risk. First, three subgroups of women expected a priori to be at higher risk of anal cancer were selected: (1) women with histologically proven cervical CIN3+ diagnosed from enrollment into CVT or its LTFU and ending at the year 11 study visit, (2) women with prevalent anal HPV16 infection at the 4‐year visit in CVT or at enrollment into the UCG, and (3) women with prevalent cervical HPV16 infection, given the elevated risk of anal cancer,^15^ at the 4‐year visit in CVT or at enrollment into the UCG. For comparison, three groups of women expected to be at lower risk of anal cancer were additionally selected: 4) women with non‐16 carcinogenic anal HPV (18/31/33/35/39/45/51/52/56/58/59) infection at the 4‐year visit in CVT or enrollment into the UCG and referred to this group as anal high‐risk (HR) HPV, (5) women with anal low‐risk (LR) HPV (6/11/34/40/42/43/44/53/54/66/68/73/70/74) infection at the 4‐year visit in CVT or enrollment into the UCG, and (6) a random sample of women who were anal HPV negative for any of the tested types. For these latter three comparison groups, as a cost‐saving measure, we limited selection to women older than 26 years because of established increasing risk of anal disease with increasing age. Figure 1 describes the study population inclusion and exclusion criteria for the anal substudy nested in CVT and referral to high‐resolution anoscopy (HRA) and is used as a guide for the sequence of events when presenting our results.

### 2.2 Follow‐up protocol

Women who participated in the anal substudy were scheduled for annual visits over three additional years to collect anal samples for HPV testing and anal cytology, cervical samples for HPV testing, and blood for immunological assays for a total of five possible study visits. For their safety prior to study exit (see below), those who were anal HPV positive at their last study visit were invited to one additional anal study visit at year 6 (i.e., 10 to 11 years since original randomization for women in the main trial; 2016–2017) of the anal substudy.

Anal cytology was evaluated at four of the five study visits (n.b. anal samples from the baseline visit were only tested for anal HPV); those with any high‐grade abnormal cytologic interpretations at any time during the study were immediately scheduled for HRA with biopsy of suspicious lesions and subsequent treatment, based on histologic findings. Treatment for abnormal lesions included topical application of trichloroacetic acid or ablation with hyfrecation. Follow‐up visits were conducted to ensure adequate treatment of lesions with additional biopsies, if needed. At the final study visit, an exit algorithm was applied to ensure women did not have more than average risk of anal disease to ensure the safe release of study participants and quantify anal disease burden (Figure S1). Women with anal HPV16/18/45 infection which had not cleared based on their final study visit were also referred to HRA, as HPV testing at the end of the study only detected HPV types 16/18/45.

### 2.3 HPV testing

Two HPV testing approaches were applied in this anal substudy, one for determining inclusion eligibility and another for clinical management during follow‐up. To select participants into the anal substudy, HPV genotyping results were based on the SPF_10_ polymerase chain reaction (PCR) primer system and DNA enzyme immunoassay detection of amplimers (DEIA, DDL Diagnostic Laboratory, Delft, Netherlands). Those positive for HPV were genotyped using the line probe assay (LiPA_25_)(SPF_10_PCR/LiPA_25_ HPV genotyping assay system, version 1, Labo Bio‐medical Products, Rijswijk, Netherlands).^16^ LiPA25 detects 25 HPV genotypes, including carcinogenic (HPV16/18/31/33/35/39/45/51/52/56/58/59) and low‐risk (6/11/34/40/42/43/44/53/54/66/68/73/70/74) types. We refer to HPV16 and non‐16 carcinogenic HPV when describing selection criteria, HPV16/18/45 during follow‐up for referral to HRA, and carcinogenic HPV when HPV16 is included with other high‐risk types in a broader context.

During the anal substudy follow‐up, anal swab samples were analyzed using a mixture of genotyping tests, with DDL used more prominently at the beginning of the study and APTIMA® (an mRNA test which only detects HPV types 16/18/45) used later. In a small sample of 247 specimens with both DDL and APTIMA results, DDL captured six HPV16 infections, of which APTIMA identified two, while there were no instances in which APTIMA identified an infection that DDL did not. The better sensitivity for DDL, which was used earlier for identifying inclusion criteria, may have implications for referral for assessment of a final study visit.

### 2.4 Cytology interpretation, referral to anoscopy and biopsy

Anal cytology slides were screened by specifically trained cytotechnologists and were interpreted by one trained local cytopathologist, using the Bethesda classification system. All the slides were additionally interpreted by an expert cytopathologist with experience in the interpretation of anal cytology and blind to the diagnosis from Costa Rica. For those with a cytologic result of HSIL, HRA was performed by a single expert clinician. At HRA, lesions suspicious for HSIL were biopsied for confirmation, along with anal cytology and HPV determination.^17^, ^18^, ^19^ Biopsies were interpreted by one pathologist in Costa Rica and one international expert. Treatment decisions were based on the international expert interpretation. Biopsy‐proven anal HSIL were then treated and monitored for recurrence using HRA based on the availability of the expert clinician.

### 2.5 Questionnaire, specimen collection

Questions about income and household facilities were only asked once during the study at the initial enrollment visit. Education was asked both at enrollment and again at the CVT year 4 visit. A risk factor questionnaire was administered at each visit, including questions regarding marital status, menstrual history, sexual and reproductive health, use of contraceptives, and smoking history.^20^ The sexual partner data was only asked at the first visit at which a woman reported being monogamous. Among women who reported a history of being sexually monogamous, additional questions were asked regarding their sexual partner, including age, education, circumcision, sexual history, and smoking history.

Among sexually active women (based on history of vaginal intercourse), personnel trained by world experts collected two anal specimens by inserting a dry swab 3–4 cm into the anal canal, rotating once, and removing the swab while continuing to rotate with gentle pressure against the wall of the anal canal. For study visits following the year four inclusion assessment visit, two anal swabs were collected and immediately placed in 1 mL of PreservCyt (Hologic, Marlborough, Massachusetts). One sample was flash frozen in vapor phase liquid nitrogen (−130 to −150°C) and the other was stored in coolers at 20°C. Then participants performed self‐sampling for collection of exfoliated cervical cells that were then placed in 20 mL of PreservCyt medium and stored in coolers at 20°C.

### 2.6 Statistical analysis

We conducted analyses by treating each of the six inclusion criteria as separate, but non‐mutually exclusive cohorts. For example, we first describe women who met the inclusion criteria based on anal HPV16 infection at the assessment visit, including those who met other inclusion criteria such as ever having a diagnosis of CIN3+. We then describe associations with meeting this selection criteria by estimating univariate prevalence ratios (PR) and 95% confidence intervals (CI) after excluding women with missing information, as not all women with cervical HPV testing had anal HPV results. We then describe the women who were included in the anal substudy, after selection and recruitment, and provide a description of all women in CVT with a year 4 visit for comparison. Finally, for women in the anal substudy, we estimated univariate PRs and 95% CIs for associations with referral to HRA using univariate regression.

## 3 RESULTS

### 3.1 Prevalence of eligibility criteria for inclusion in the anal substudy

We describe the prevalence of meeting eligibility criteria from among women in CVT (Figure 1) based on results from the year 4 study visit (Figure 1, Box 1). From among 10,302 women in CVT, there were 9,416 women (6580 from the main trial and 2836 from the UCG) who had anal and/or cervical HPV specimens collected at year 4 after initial randomization of women, and 4,905 had anal HPV test results.

### 3.2 Descriptive characteristics of the anal subcohort

We describe the characteristics of the cohort of 1,174 women with year 4 anal HPV test results included in our study according to each inclusion as well as a comparison of those women in CVT who were not included in our anal subcohort (Table 1). For example, among 93 women included in the anal substudy who had a prevalent anal HPV16 infection, only 18% received any dose of the HPV vaccine, while 35% of women included as anal HPV negative random controls had received at least one dose. Among those with a history of CIN3+, 105 (30.2%) were from the original vaccinated study arm. The random controls were similar to the full CVT cohort of women who had anal HPV testing at the year 4 visit and will serve as an essential comparison group in future analyses.

**TABLE 1:** Characteristics of women in the anal substudy nested in the Costa Rica HPV Vaccine Trial according to inclusion criteria at year 4.

		Women included in the anal cohort according to inclusion criteria						All women in CVT with a year 4 visit and not in the anal cohort
	All women included in the anal cohort	Anal HPV16+ at baseline visit	Ever CIN3+ histology	Cervical HPV16+ at baseline visit	Anal non‐16 carcinogenic HPV and 26+ years old at baseline	Anal low‐risk HPV+ and 26+ years old at baseline	Random control (anal HPV−, 26+ years old)	
	*N* (%)	*n* (%)	*n* (%)	*n* (%)	*n* (%)	*n* (%)	*n* (%)	
*N* eligible (row %)	1174^a^	93 (9.3)	348 (29.6)	236 (20.1)	364 (41.8)	295 (33.9)	227 (26.1)	8242
Study design features								
Study arm								
Cervarix	391 (33.3)	17 (18.3)	105 (30.2)	27 (11.4)	122 (33.5)	137 (46.4)	80 (35.2)	2890 (35.1)
Havrix	436 (37.1)	48 (51.6)	120 (34.5)	85 (36.0)	143 (39.3)	120 (40.7)	74 (32.6)	2863 (34.7)
UCG	347 (29.6)	28 (30.1)	123 (35.3)	124 (52.5)	99 (27.2)	38 (12.9)	73 (32.2)	2489 (30.2)
Vaccination status								
Unvaccinated	783 (66.7)	76 (81.7)	243 (69.8)	209 (88.6)	242 (66.5)	158 (53.6)	147 (64.8)	5352 (64.9)
1 or 2 dose	79 (6.7)	3 (3.2)	30 (8.6)	3 (1.3)	31 (8.5)	23 (7.8)	13 (5.7)	475 (5.8)
3 doses	312 (26.6)	14 (15.1)	75 (21.6)	24 (10.2)	91 (25.0)	114 (38.6)	67 (29.5)	2415 (29.3)
Age at year 4								
Median (IQR)	27 (26–29)	26 (24–28)	27 (25–28)	26 (24–28)	28 (27–29)	28 (27–29)	28 (27–29)	25 (24–28)
Sexual history								
Lifetime number of vaginal sex partners								
Unknown	4 (0.3)	0 (0.0)	2 (0.6)	0 (0.0)	1 (0.3)	1 (0.3)	1 (0.4)	17 (0.2)
0	2 (0.2)	N/a	2 (0.6)	0 (0.0)	N/a	N/a	N/a	451 (5.5)
1	206 (17.5)	17 (18.3)	57 (16.4)	36 (15.3)	41 (11.3)	30 (10.2)	70 (30.8)	2291 (27.8)
2	250 (21.3)	13 (14.0)	76 (21.8)	55 (23.3)	61 (16.8)	55 (18.6)	50 (22.0)	1665 (20.2)
3+	712 (60.6)	63 (67.7)	211 (60.6)	145 (61.4)	261 (71.7)	209 (70.8)	106 (46.7)	3818 (46.3)
Age at first vaginal sex								
Median (IQR)	17 (15–18)	17 (15–18)	16 (15–18)	17 (15–18)	17 (15–18)	17 (15–18)	17 (15–18)	17 (15–19)
Age at first anal sex								
Median (IQR)	24 (21–26)	23 (21–26)	23 (20–25)	22 (20–25)	25 (22–27)	25 (22–27)	24.5 (20–27)	23 (20–25)
Lifetime number of anal sex partners								
Unknown	87 (7.4)	2 (2.2)	46 (13.2)	56 (23.7)	8 (2.2)	2 (0.7)	0 (0.0)	1069 (13.0)
0	789 (67.2)	63 (67.7)	225 (64.7)	143 (60.6)	230 (63.2)	189 (64.1)	179 (78.9)	5942 (72.1)
1	229 (19.5)	20 (21.5)	59 (17.0)	30 (12.7)	94 (25.8)	77 (26.1)	40 (17.6)	1048 (12.7)
2+	69 (5.9)	8 (8.6)	18 (5.2)	7 (3.0)	32 (8.8)	27 (9.2)	8 (3.5)	183 (2.2)
Demographics								
Marital status								
Unknown	2 (0.2)	0 (0.0)	1 (0.3)	0 (0.0)	1 (0.3)	1 (0.3)	0 (0.0)	4 (0.0)
Married/living with partner	767 (65.3)	54 (58.1)	217 (62.4)	146 (61.9)	235 (64.6)	179 (60.7)	175 (77.1)	5337 (64.8)
Single	303 (25.8)	30 (32.3)	100 (28.7)	72 (30.5)	93 (25.5)	82 (27.8)	34 (15.0)	2465 (29.9)
Divorced/separate/widowed	102 (8.7)	9 (9.7)	30 (8.6)	18 (7.6)	35 (9.6)	33 (11.2)	18 (7.9)	436 (5.3)
Education								
Unknown	6 (0.5)	1 (1.1)	3 (0.9)	2 (0.8)	1 (0.3)	1 (0.3)	1 (0.4)	63 (0.8)
≤6 y	426 (36.3)	24 (25.8)	123 (35.3)	65 (27.5)	134 (36.8)	107 (36.3)	94 (41.4)	2429 (29.5)
7–9 y	258 (22.0)	25 (26.9)	84 (24.1)	66 (28.0)	81 (22.3)	65 (22.0)	40 (17.6)	1560 (18.9)
≥10 y+ technical	254 (21.6)	20 (21.5)	66 (19.0)	49 (20.8)	80 (22.0)	66 (22.4)	54 (23.8)	2121 (25.7)
University	230 (19.6)	23 (24.7)	72 (20.7)	54 (22.9)	68 (18.7)	56 (19.0)	38 (16.7)	2069 (25.1)
Smoking history								
Unknown	2 (0.2)	0 (0.0)	1 (0.3)	0 (0.0)	1 (0.3)	1 (0.3)	0 (0.0)	10 (0.1)
Never (%)	911 (77.6)	72 (77.4)	266 (76.4)	180 (76.3)	276 (75.8)	221 (74.9)	194 (85.5)	6812 (82.6)
Ever (%)	261 (22.2)	21 (22.6)	81 (23.3)	56 (23.7)	87 (23.9)	73 (24.7)	33 (14.5)	1420 (17.2)

^a^ 4 women who were included in the anal substudy did not have year 4 data collected but were included based on a diagnosis of CIN3+.

### 3.3 Factors associated with each inclusion criteria for the anal substudy

Among the 4905 women with anal HPV test results from the year 4 visit, 145 were positive for anal HPV16 (3.0%) (Table 2). Among the 2909 women older than 26 years of age at the visit used to assess eligibility for inclusion in the anal substudy with anal HPV results, 509 (17.5%) were positive for an anal non‐16 carcinogenic HPV infection and 665 (22.9%) were positive for an anal LR‐HPV infection, leaving 1893 (65.1%) who had neither carcinogenic nor anal LR‐HPV infection. Of the 9416 women with follow‐up in CVT, there were 326 (3.5%) that had a diagnosis of CIN3+ at or before the baseline visit, and an additional 194 women were diagnosed with CIN3+ after the baseline visit and became eligible for follow‐up in the anal substudy. Of 9404 women with cervical HPV results from the year 4 visit, 404 (4.3%) had a prevalent cervical HPV16 infection.

**TABLE 2:** Descriptive characteristics and risk factors for meeting inclusion criteria for the anal follow‐up study among women in the Costa Rica HPV Vaccine Trial based on information from the year 4 study visit.

	Univariate prevalence ratios (95% CI)											
	Anal HPV16+ at baseline visit		CIN3+ histology		Cervical HPV16+ at baseline visit		Anal non‐16 carcinogenic HPV+ and 26+ years old at baseline		Anal low‐risk HPV+ and 26+ years old at baseline		Random control (anal HPV−, 26+ years old)	
Total *N*	145/4905 (3.0%)		298^a^/9416 (3.2%)		404/9404 (4.3%)		509/2909 (17.5%)		665/2909 (22.9%)		1893/2909 (65.1%)	
*Study design features*												
	*n* (%)	PR (95%CI)	*n* (%)	PR (95%CI)	*n* (%)	PR (95%CI)	*n* (%)	PR (95%CI)	*n* (%)	PR (95%CI)	*n* (%)	PR (95%CI)
Anal HPV16 at baseline												
Positive	–	–	17 (5.7)	*3.9* (*2.4–6.3*)	72 (17.8)	*13.8* (*10.7–17.9*)	43 (8.4)	*3.1* (*2.3–4.3*)	29 (4.4)	*1.6* (*1.1–2.3*)	0 (0.0)	N/a
Negative	–	–	281 (94.3)	Ref	332 (82.2)	Ref	466 (91.6)	Ref	636 (95.6)	Ref	1893 (100.0)	Ref
History of CIN3+ histology at baseline												
Ever	17 (11.7)	*3.0* (*1.8–4.9*)	–	–	61 (15.1)	*5.4* (*4.1–7.1*)	33 (6.5)	1.3 (0.9–1.9)	42 (6.3)	1.3 (0.9–1.7)	80 (4.2)	0.8 (0.7–1.0)
Never	128 (88.3)	Ref	–	–	343 (84.9)	Ref	476 (93.5)	Ref	623 (93.7)	Ref	1813 (95.8)	Ref
Cervical HPV16 at baseline												
Positive	72 (49.7)	*20.6* (*14.9–28.5*)	61 (20.5)	*5.7* (*4.3–7.6*)	–	*–*	30 (5.9)	*1.5* (*1.1–2.2*)	32 (4.8)	1.2 (0.9–1.8)	51 (2.7)	*0.7* (*0.5–0.9*)
Negative	73 (50.3)	Ref	237 (79.5)	Ref	–	–	479 (94.1)	Ref	633 (95.2)	Ref	1842 (97.3)	Ref
Anal non‐16 carcinogenic HPV at baseline and older than 26 years												
Positive	43 (29.7)	*3.6* (*2.5–5.2*)	33 (11.1)	*2.2* (*1.5–3.1*)	30 (7.4)	1.4 (1.0–2.0)	–	–	185 (27.8)	*1.8* (*1.5–2.2*)	0 (0.0)	N/a
Negative	102 (70.3)	Ref	265 (88.9)	Ref	374 (92.6)	Ref	–	–	480 (72.2)	Ref	1893 (100.0)	Ref
Anal low‐risk HPV+ and 26+ years old at baseline												
Positive	29 (20.0)	*1.6* (*1.1–2.4*)	42 (14.1)	*2.2* (*1.6–3.0*)	32 (7.9)	1.1 (0.8–1.6)	185 (36.3)	*1.9* (*1.6–2.3*)	–	–	0 (0.0)	N/a
Negative	116 (80.0)	Ref	256 (85.9)	Ref	372 (92.1)	Ref	324 (63.7)	Ref	–	–	1893 (100.0)	Ref
Random control (anal HPV−, 26+ years old)												
Yes	0 (0.0)	N/a	80 (26.8)	*1.5* (*1.1–1.9*)	51 (12.6)	*0.6* (*0.4–0.8*)	0 (0.0)	N/a	0 (0.0)	N/a	–	–
No	145 (100.0)	Ref	218 (73.2)	Ref	353 (87.4)	Ref	509 (100.0)	Ref	665 (100.0)	Ref	–	–
Study arm												
Cervarix	27 (18.6)	Ref	114 (38.3)	Ref	45 (11.1)	Ref	184 (36.1)	Ref	267 (40.2)	Ref	766 (40.5)	Ref
Havrix	85 (58.6)	*3.1* (*2.0–4.8*)	152 (51.0)	1.3 (1.0–1.7)	164 (40.6)	*3.6* (*2.6–5.0*)	208 (40.9)	1.1 (0.9–1.4)	259 (38.9)	1.0 (0.8–1.2)	723 (38.2)	0.9 (0.9–1.1)
UCG^b^	33 (22.8)	*3.8* (*2.3–6.4*)	32 (10.7)	*0.3* (*0.2–0.5*)	195 (48.3)	*5.0* (*3.6–6.9*)	117 (23.0)	1.1 (0.9–1.4)	139 (20.9)	0.9 (0.8–1.1)	404 (21.3)	0.9 (0.8–1.1)
Vaccination status												
Unvaccinated	118 (81.4)	Ref	184 (61.7)	Ref	359 (88.9)	Ref	325 (63.9)	Ref	398 (59.8)	Ref	1127 (59.5)	Ref
1 or 2 dose	5 (3.4)	*0.3* (*0.1–0.8*)	31 (10.4)	*1.9* (*1.3–2.7*)	6 (1.5)	*0.2* (*0.1–0.4*)	45 (8.8)	1.3 (0.9–1.7)	43 (6.5)	1.0 (0.7–1.4)	121 (6.4)	1.0 (0.8–1.2)
3 doses	22 (15.2)	*0.3* (*0.2–0.5*)	83 (27.9)	1.0 (0.8–1.3)	39 (9.7)	*0.2* (*0.2–0.3*)	139 (27.3)	0.8 (0.7–1.0)	224 (33.7)	1.1 (0.9–1.2)	645 (34.1)	1.1 (1.0–1.2)
Age (median; range)	26 (22–32)	1.0 (0.9–1.1)	26 (21–33)	*1.1* (*1.1–1.2*)	26 (21–33)	1.0 (0.9–1.0)	28 (26–32)	1.0 (0.9–1.1)	28 (26–32)	1.0 (0.9–1.0)	28 (26–32)	1.0 (1.0–1.0)
Younger than 26 years	62 (42.8)	Ref	96 (32.2)	*Ref*	207 (51.2)	Ref	0 (0.0)	Ref	0 (0.0)	Ref	0 (0.0)	Ref
26 years or older	83 (57.2)	0.9 (0.7–1.3)	202 (67.8)	*1.9* (*1.5–2.4*)	197 (48.8)	0.8 (0.7–1.0)	509 (100.0)	N/a	665 (100.0)	N/a	1893 (100.0)	N/a

*Note*: Inclusion criteria are not mutually exclusive, allowing for women to be counted in multiple strata, and therefore, the sum of the strata is greater than the total number of women.

Abbreviations: CI, Confidence Interval; CIN3+, Cervical intraepithelial neoplasia grade 3 or worse; HPV, Human papillomavirus; PR, Prevalence Ratio; Ref, Referent group.

^a^ 28 women with CIN3+ did not have a baseline visit and were excluded from this table.

^b^ Not assessed at L0 for UCG women.

We present a description of factors for meeting each of the inclusion criteria, as criteria were not mutually exclusive. For example, of the 145 women who were anal HPV16 positive, 33 (22.8%) had a CIN3+ diagnosis for a PR of 4.2 (95%CI = 2.8–6.1) compared to women who were anal HPV16 negative. Seventy‐two (49.7%) also had a prevalent cervical HPV16 infection (PR = 20.1, 95%CI = 14.9–28.5); 43 (29.7%) were older than 26 years and also had an anal non‐16 carcinogenic HPV infection (PR = 3.6, 95%CI = 2.6–5.2); and 29 (20.0%) were older than 26 years and also had an anal LR‐HPV infection (PR = 1.6, 95%CI = 1.1–2.4) (Table 2). Women from the Havrix and UCG study arms were 3.1‐times and 3.8‐times more likely, respectively, to have a prevalent anal HPV16 infection at the year 4 visit than those who were in the Cervarix arm. Over 80% of those with a prevalent anal HPV16 infection were unvaccinated at the time of the eligibility study visit. We present associations with each inclusion criterion and sexual history and demographic characteristics in Table S1.

As some criteria were restricted to women age 26 years and older, we assessed potential bias for generalizing to younger women by describing anal HPV prevalence according to age at the baseline visit. Prevalence of any anal HPV infection was slightly less common in those 26 years and older (35%) compared to those younger than 26 years (39%) (PR = 0.90, *p* value = 0.03). However, there were no differences between groups for HPV16, carcinogenic types overall, or when comparing by grouped vaccine types (Table 3). Non‐carcinogenic anal HPV prevalence was more common in those younger than 26 years (PR = 0.87 vs. 26+ years, *p* value = 0.03) and likely explained the difference in anal HPV prevalence overall.

**TABLE 3:** Prevalence of anal HPV by type according to age at time of study visit.

HPV type	<26yo (1996 women tested)	≥26yo (2909 women tested)	Prevalence ratio (95% Confidence interval)	*p* value
Any	775 (38.8)	1016 (34.9)	0.9 (0.8–1.0)	*0.03*
Carcinogenic types	427 (21.4)	549 (18.9)	0.9 (0.8–1.0)	0.05
16	62 (3.1)	83 (2.9)	0.9 (0.7–1.3)	0.61
18	33 (1.7)	44 (1.5)	0.9 (0.6–1.4)	0.70
31	45 (2.3)	73 (2.5)	1.1 (0.8–1.6)	0.57
33	20 (1.0)	20 (0.7)	0.7 (0.4–1.3)	0.23
35	23 (1.2)	31 (1.1)	0.9 (0.5–1.6)	0.78
39	58 (2.9)	65 (2.2)	0.8 (0.5–1.1)	0.15
45	21 (1.1)	37 (1.3)	1.2 (0.7–2.1)	0.49
51	115 (5.8)	120 (4.1)	0.7 (0.6–0.9)	*0.01*
52	97 (4.9)	120 (4.1)	0.8 (0.7–1.1)	0.23
56	60 (3.0)	59 (2.0)	0.7 (0.5–1.0)	*0.03*
58	24 (1.2)	56 (1.9)	1.6 (1.0–2.6)	0.05
59	16 (0.8)	24 (0.8)	1.0 (0.6–1.9)	0.93
Low‐risk types	398 (19.9)	502 (17.3)	0.9 (0.8–1.0)	*0.03*
6	60 (3.0)	67 (2.3)	0.8 (0.5–1.1)	0.13
11	8 (0.4)	9 (0.3)	0.8 (0.3–2.0)	0.59
34	3 (0.2)	4 (0.1)	0.9 (0.2–4.1)	0.91
40	8 (0.4)	15 (0.5)	1.3 (0.6–3.0)	0.56
42	4 (0.2)	3 (0.1)	0.5 (0.1–2.3)	0.38
43	15 (0.8)	25 (0.9)	1.1 (0.6–2.2)	0.68
44	40 (2.0)	54 (1.9)	0.9 (0.6–1.4)	0.72
53	90 (4.5)	106 (3.6)	0.8 (0.6–1.1)	0.14
54	39 (2.0)	57 (2.0)	1.0 (0.7–1.5)	0.99
66	78 (3.9)	85 (2.9)	0.8 (0.6–1.0)	0.06
68/73	48 (2.4)	51 (1.8)	0.7 (0.5–1.1)	0.12
70	48 (2.4)	67 (2.3)	1.0 (0.7–1.4)	0.82
74	61 (3.1)	83 (2.9)	0.9 (0.7–1.3)	0.69
4‐valent	148 (7.4)	192 (6.6)	0.9 (0.7–1.1)	0.29
2‐valent	92 (4.6)	123 (4.2)	0.9 (0.7–1.2)	0.53
9‐valent	302 (15.1)	421 (14.5)	1.0 (0.8–1.1)	0.56
Uncharacterized	123 (6.2)	163 (5.6)	0.9 (0.7–1.2)	0.43

*Note*: 2‐valent: 16, 18; 4‐valent: 6, 11, 16, 18; 9‐valent: 6, 11, 16, 18, 31, 33, 45, 52, 58. Of the 1996 women younger than 26 years, the most common HPV type in our study was HPV 51 (5.8%), followed by HPV 52 (4.9%), 53 (4.5%), 66 (3.9%), and then 16 and 74 (both 3.1%) and there were 123 (6.2%) with at least one uncharacterized HPV type. Among the 2909 women 26 years or older the most common types were HPV 51 and 52 (both 4.1%), 53 (3.6%), 66 (2.9%), and 16 and 74 (both 2.9%) with 163 (5.6%) with at least one uncharacterized HPV type.

### 3.4 Recruitment and participation of women in the anal substudy

We describe the selection and participation of eligible women (Figure 1, Box 2) in the anal substudy (Figure 1, Box 3). There were an additional 141 diagnoses of CIN3+ after the year 4 visit for a total of 2021 who were invited to participate in the anal substudy. There were 1178 women who accepted participation for an overall acceptance rate of 64% (Table 4). Acceptance of participation in the anal substudy ranged from 49% for women invited based on a low‐risk anal HPV infection to 81% of women who were invited as random controls based on a negative anal HPV test. In total, we carried out 3033 study visits following the baseline assessment visit among the 1178 women with a median follow‐up time of 66.9 months. The median follow‐up time was similar across each group based on inclusion criteria. As of December 31, 2023, there have been 58 women who have attended an anoscopy visit. Of the 1178 final sample of women who were included in the anal substudy based on non‐mutually exclusive selection criteria, 93 (7.9%) had a prevalent anal HPV16 infection, 352 (29.9%) had a prior diagnosis of CIN3+, 236 (20.0%) had a prevalent cervical HPV16 infection, 364 (30.9%) had a prevalent anal non‐16 carcinogenic HPV infection, 295 (25.0%) had a prevalent anal LR‐HPV infection, and 227 (19.3%) were recruited as controls based on a negative test for prevalent anal HPV infection.

**TABLE 4:** Sampling fractions, participation proportions, visit history, and referral to HRA according to inclusion criteria from the Costa Rica HPV Vaccine Trial and anal substudy.

Inclusion criteria	Total eligible	Target sampling fraction	Approached (%)	Accepted (%: participation proportion)	Overall participation (%)	1 anal visit	2 anal visits	3 anal visits	4 anal visits	Total number of visits	Median follow‐up time in months (IQR)	Referred to HRA (%)	Attended HRA
Anal HPV16+ at baseline visit	145	100%	126 (86.9)	93 (73.8)	93/145 (64.1)	14	15	47	17	253	69.7 (63.6–100.9)	17 (18.3)	14
Ever CIN3+ histology	520	100%	499 (96.0)	352 (70.5)	352/520 (67.7)	60	100	160	32	868	67.1 (62.1–73.7)	25 (7.1)	21
Cervical HPV16+ at baseline visit	404	100%	380 (94.1)	236 (62.1)	236/404 (58.4)	27	63	118	28	619	67.9 (62.3–74.6)	15 (6.4)	14
Anal non‐16 carcinogenic HPV+ and 26+ years old at baseline	509	100%	455 (89.4)	364 (80.0)	364/509 (71.5)	42	77	215	30	961	66.5 (61.3–72.2)	23 (6.3)	18
Anal low‐risk HPV+ and 26+ years old at baseline	665	50%	599 (90.1)	295 (49.2)	295/665 (44.4)	33	70	170	22	771	65.8 (61.0–70.9)	13 (4.4)	11
Random control (anal HPV−, 26+ years old)	1893	299^a^	279 (14.7)	227 (81.4)	227/1893 (12.0)	27	62	115	23	588	67.1 (60.6–73.6)	11 (4.8)	9
Total	3483		1829 (52.5)	1178 (64.4)	1178/3483 (33.8)	156	298	615	109	3033	66.9 (61.6–73.0)	71 (6.0)	58

^a^ A target sample size was designed for random controls as opposed to the target fraction for other inclusion criteria.

### 3.5 Follow‐up and referral to anoscopy

For the 1,178 women included in the anal substudy (Figure 1, Box 3), describe factors associated with referral to HRA (Figure 1, Box 4). Of the 1,178 women in our study, 883 (75.0%) had normal anal cytology results and 925 (78.5%) were anal HPV positive at all study visits (over 2.5 visits on average). There were 182 women who cleared a prevalent or incident anal HPV16/18/45 infection and 220 women with either a low‐grade anal lesion based on cytology result or atypical squamous cell of unknown significance (Figure 1). There were 71 women referred to HRA, of which 28 were based on a high‐grade lesion anal cytology result and 45 on anal HPV16/18/45 positivity at their last visit (Figure 1, Box 4). The proportion of women referred to HRA based on each inclusion criterion using the recruitment assessment visit for eligibility into the anal substudy was 18.3% for those with a prevalent anal HPV16 infection, 7.2% for women with a prior CIN3+ by initiation of the anal substudy, 6.4% of women with a prevalent cervical HPV16 infection, 6.3% of women with a prevalent anal non‐16 carcinogenic HPV infection, 4.4% for prevalent anal LR‐HPV infection, and 4.8% for women recruited as random controls.

### 3.6 Factors associated with meeting referral criteria for anoscopy

Compared to women in the anal substudy who were included as random controls, those with a prevalent anal HPV16 infection were 3.6‐times (95%CI = 1.7–7.7) more likely to eventually be referred to HRA (Table 5). Women from the UCG study arm were more than twice (95%CI = 1.2–3.8) as likely to be referred to HRA compared to women in the original vaccinated arm. Each additional year in age at first vaginal intercourse was associated with a 10% decrease in the proportion of women referred to HRA (95%CI = 0.80–0.97) and women with no anal sex partners in their lifetime were half as likely to be referred to HRA compared to women with one lifetime anal sex partner. Of 524 women without any prior cervical non‐16 carcinogenic HPV infection, 15 (2.9%) were referred to HRA, representing a PR for referral to HRA of 0.36 (95%CI = 0.19–0.69) compared to women with a history of any non‐16 carcinogenic HPV infection. There were not notable associations with marital status nor smoking, and an unclear direction of association for education, for which women with 7 to 9 years of education were nearly twice as likely to be referred to HRA compared to women with 6 years of education or less.

**TABLE 5:** Descriptive characteristics of women at year 4 in the Costa Rica Vaccina Trial (CVT) study with any anal cytology results by referral status to high‐resolution anoscopy (HRA).

	Referred to HRA	Women in CVT with a year 4 visit and never referred to HRA	Univariate prevalence ratio (95% CI)
	*N* (column %; 95% CI)	*N* (column %; 95% CI)	
Total *N*	71	1103^a^	
Study design features			
Inclusion criteria			
Anal HPV16+ at baseline visit	17	76	*3.6* (*1.7–7.7*)
Ever CIN3+ histology	25	323	1.4 (0.7–2.9)
Cervical HPV16+ at baseline visit	15	221	1.3 (0.6–2.7)
Anal non‐16 carcinogenic HPV and 26+ years old at baseline	23	341	1.3 (0.6–2.6)
Anal low‐risk HPV+ and 26+ years old at baseline	13	282	0.9 (0.4–2.0)
Random control (anal PCR−, 26+ years old)	11	207	Ref
Study arm			
Cervarix	17	374	Ref
Havrix	22	414	1.2 (0.6–2.2)
UCG	32	315	*2.1* (*1.2–3.8*)
Vaccination status			
Unvaccinated	54	729	Ref
1 or 2 dose	5	74	0.9 (0.4–2.3)
3 doses	12	300	0.6 (0.3–1.0)
Age at year 4 visit			
Median (IQR; range)	27	27	1.0 (0.9–1.1)
Sexual history			
Lifetime number of vaginal sex partners			
Unknown	0	4	N/a
0	0	2	N/a
1	7	199	Ref
2	11	239	1.3 (0.5–3.3)
3+	53	659	2.2 (1.0–4.8)
Age at first vaginal sex			
Median (IQR; range)	16	17	*0.9* (*0.8–1.0*)
Age at first anal sex			
Median (IQR; range)	24.5	24	1.0 (0.9–1.1)
Lifetime number of anal sex partners			
Unknown	3	84	0.3 (0.1–1.1)
0	40	749	*0.5* (*0.3–0.8*)
1	23	206	Ref
2+	5	64	0.7 (0.3–1.9)
Any prior cervical HR‐HPV+ (%)			
Negative	*15*	*509*	*0.4* (*0.2–0.7*)
HPV 16+	15	191	*1.9* (*1.0–3.6*)
HPV18/45+	*15*	*136*	*2.8* (*1.5–5.3*)
Demographics			
Marital status			
Unknown	0	2	N/a
Married/living with partner	48	719	Ref
Single	18	285	0.9 (0.6–1.6)
Divorced/separate/widowed	5	97	0.8 (0.3–2.0)
Education			
Unknown	0	6	N/a
≤6 y	21	405	Ref
7–9 y	25	233	*2.0* (*1.1–3.5*)
≥10 y + technical	9	245	0.7 (0.3–1.6)
University	16	214	1.4 (0.7–2.7)
Smoking history			
Unknown	0	2	N/a
Never	52	859	Ref
Ever	19	242	1.3 (0.8–2.2)

*Note*: Referral to HRA was based on anal HSIL cytology results at any point during the anal substudy or anal HPV positivity at participant's last study visit. Infection with human papillomavirus is the primary cause of anal cancer, which predominantly affects women. There is limited evidence to inform effective anal cancer screening strategies for women without HIV. Here, the authors designed a study nested within the Costa Rica HPV Vaccine Trial to understand the natural history of anal HPV infection and to identify high‐risk women for high‐resolution anoscopy referral. From 10,302 women, 1178 women were followed over 3033 annual visits and 71 referred to HRA. The findings will inform our understanding of the natural history of anal HPV infection and inform guidelines for anal cancer screening programs.

Abbreviation: CI, Confidence interval.

^a^ Note that 4 women missed their year 4 visit and were excluded from this table but are included in preceding analyses.

## 4 DISCUSSION

To define the natural history of anal HPV and related disease among young, HIV‐negative women, we created an anal natural history study within an existing study infrastructure originally designed for a clinical trial of the bivalent HPV vaccine. By leveraging a priori inclusion criteria, we have essentially allowed for the assessment of multiple cohorts while maintaining the potential to generalize to a larger population. In this manuscript, we describe the study rationale and methods for following 1,178 women with annual study visits over a median follow‐up of 5 years. These first findings show that among those 1,178 women, approximately 6% met criteria that merited HRA based on annual anal HPV testing with cytology. We further present results of implementing a risk‐based referral to HRA for groups of these HIV‐negative women, including those with a prevalent anal HPV infection, a diagnosis of CIN3+, and those with a prevalent cervical HPV16 infection. Importantly, these groups represent categories of risk defined by screening guidelines as having elevated rates of anal cancer.^21^ Our description of factors associated with meeting these inclusion criteria is consistent with previous study showing an association between the number of lifetime partners and the prevalence of anal HPV and anal disease.^20^, ^22^

Evidence‐based anal cancer screening guidelines now exist in response to recent findings from the ANCHOR trial, including the use of cytology and carcinogenic HPV testing for triage for referral to HRA.^21^ Our follow‐up and referral algorithm aligns with prescribed management strategies for cytologic and carcinogenic HPV results for populations defined as risk category B by the International Anal Neoplasia Society (e.g., women with cervical precancer and those with persistent cervical carcinogenic HPV), as well as women who would be classified as the general population.^21^, ^23^ Although we applied these screening and referral strategies to a population that is younger than currently recommended, it may serve as an important comparator for justifying a minimum age to begin screening.

As women with lower genital tract precancer are known to be at elevated risk for anal cancer,^6^, ^24^, ^25^ we further describe the occurrence of both anal and cervical HPV16 among women with a prior CIN3+. A prior study of women undergoing excisional treatment for cervical neoplasia estimated that as much as 62% had prevalent anal HPV infections.^15^ In our study population, we found that half of the women with anal HPV16 also had a prevalent cervical HPV16 infection and 23% ever had a diagnosis of CIN3+. From this group of women with CIN3+, we build on a previously reported subset of women from this study with the addition of women in the UCG arm.^20^ Previous estimates of anal HSIL in HIV‐negative women have ranged from 0 to 3% based on a systematic review.^6^ However, age was unaccounted for and likely a determining factor considering the age distribution of anal cancer and anal precancer incidence.^3^, ^26^ HPV infection is estimated to occur roughly two decades prior to anal cancer^27^; therefore, our study of young immunocompetent women ages 21 to 32 years may represent an ideal age range to understand HPV infection despite the overall low referral to HRA.

While notable effort was made to maximize the efficient use of resources and considering that these women had already committed several years to involvement in the original clinical trial, we acknowledge imperfect sampling. For example, we only initiated anal sample collection in the UCG midway through its enrollment; we only included women older than 26 in some of the sampling frames. Future study is necessary to account for our sampling framework and selection criteria to generalize to the larger population. Although the referral algorithm for HRA was uniform for all women, the performance of anal cytology for predicting anal HSIL has been shown to have limited sensitivity and specificity.^28^

In conclusion, we provide a comprehensive overview of the design, study population, and sampling framework of a unique resource to investigate the natural history and clinical outcomes related to anal cancer in a cohort of vaccinated and unvaccinated young, healthy women. This study encompasses several important potential cohorts for investigating and describing anal HPV and related dysplasia. Future study will describe the natural history of anal HPV in this population of young, vaccinated and unvaccinated women in order to inform screening strategies and primary prevention of anal cancer. We will describe factors associated with a biopsy‐confirmed HSIL for those women who have been referred to HRA, as well as the testing performance and characteristics of anal HPV and cytology testing compared to biopsy. As some women remain in care for follow‐up of their lesions, we will also describe the clinical trajectories of these women with biopsy‐confirmed HSIL and treatment.

## AUTHOR CONTRIBUTIONS

**Cameron B. Haas:** Conceptualization; investigation; writing – original draft; methodology; formal analysis. **Rebeca Ocampo:** Investigation; data curation; writing – review and editing; project administration. **Danping Liu:** Methodology; writing – review and editing; formal analysis. **Michael Zúñiga:** Writing – review and editing; project administration; resources. **Diego Guillen:** Writing – review and editing; project administration; resources; data curation. **Megan A. Clarke:** Writing – review and editing; methodology; investigation; formal analysis. **Loretto J. Carvajal:** Writing – review and editing. **Allan Hildesheim:** Conceptualization; methodology; writing – review and editing; project administration. **John Schussler:** Formal analysis; writing – review and editing. **Mónica Sierra:** Project administration; writing – review and editing. **Teresa M. Darragh:** Writing – review and editing; formal analysis. **Joel M. Palefsky:** Investigation; writing – review and editing; resources; formal analysis; methodology. **Carolina Porras:** Investigation; resources; data curation; writing – review and editing. **Aimée R. Kreimer:** Investigation; writing – review and editing; funding acquisition; project administration; supervision; formal analysis; methodology. **Rolando Herrero:** Investigation; writing – review and editing; methodology; project administration; formal analysis.

## FUNDING INFORMATION

The Costa Rica HPV Vaccine Trial is a long‐standing collaboration between investigators in Costa Rica and the US National Cancer Institute (NCI). The trial is sponsored and funded by the NCI (contract N01‐CP‐11005) with funding support from the National Institutes of Health Office of Research on Women's Health. GlaxoSmithKline Biologicals provided vaccine and support for aspects of the trial associated with regulatory submission needs of the company under a Clinical Trials Agreement (FDA BB‐IND 7920) during the four‐year randomized blinded phase of the study, but had no role in the study design, data collection, data analysis, data interpretation, or writing of the report. The NCI and Costa Rica investigators are responsible for the design and conduct of the study; collection, management, analysis, and interpretation of the data; and preparation of the manuscript. All authors have access to the raw data.

## CONFLICT OF INTEREST STATEMENT

Joel M. Palefsky reports institutional grant support from Roche Diagnostics and Atila Biosystems. He has received personal compensation for consulting for Vir Biotechnologies, Merck, Asieris Pharmaceuticals, Spotlight Therapeutics, Abbott, and GSK. He has stock options in Virion Therapeutics. All other authors have no conflicts of interest to disclose.

## ETHICS STATEMENT

All women provided written, informed consent. Research activity for CVT (ClinicalTrials.gov ID: NCT05237947) was approved by Institutional Review Boards of Instituto Costarricense de Investigación y Enseñanza en Nutritión y Salud in Costa Rica (#CEC‐HCB‐E004‐2024) and the United States National Cancer Institute (#09‐C‐N106) (Bethesda, MD).

## THE COSTA RICA HPV VACCINE TRIAL (CVT) GROUP

Bernal Cortés, Paula González (deceased), Rolando Herrero, Silvia E. Jiménez, Carolina Porras, Ana Cecilia Rodríguez (Agencia Costarricense de Investigaciones Biomédicas [ACIB], formerly Proyecto Epidemiológico Guanacaste, PEG, Fundación INCIENSA, San José, Costa Rica); Allan Hildesheim, Aimée R. Kreimer, Douglas R. Lowy, Mark Schiffman, John T. Schiller, Mark Sherman, Sholom Wacholder (deceased) (United States National Cancer Institute, Bethesda, MD, USA); Ligia A. Pinto, Troy J. Kemp (Leidos Biomedical Research, Inc., Frederick National Laboratory for Cancer Research, Frederick, MD, USA); Mary K. Sidawy (Georgetown University, Washington, DC, USA); Wim Quint (deceased), Leen‐Jan van Doorn, Linda Struijk (DDL Diagnostic Laboratory, Netherlands); Joel M. Palefsky, Teresa M. Darragh (University of California, San Francisco, CA, USA); Mark H. Stoler (University of Virginia, Charlottesville, VA, USA).

## Supporting information


**DATA S1.** Supporting information.

## Data Availability

A trial summary, current publications, and contact information for data access are available online: https://dceg.cancer.gov/research/who-we-study/cohorts/costa-rica-vaccine-trial. Further information is available from the corresponding author upon request.
