# Supplementary material for: Design and initial findings of a natural history study of anal HPV and associated lesions among young adult women in Costa Rica
Source: Int J Cancer. 2025 Aug 15;157(12):2603–18. doi: 10.1002/ijc.70082 (PMC12541566; doi:10.1002/ijc.70082)
Supplement: Supplementary file 1 — DATA S1. Supporting information. [file IJC-157-2603-s001.pdf]

DESIGN AND INITIAL FINDINGS OF A NATURAL HISTORY STUDY OF ANAL HPV AND ASSOCIATED LESIONS AMONG YOUNG ADULT WOMEN IN COSTA RICA

Authors: Cameron B. Haas, Rebeca Ocampo, Danping Liu, Michael Zúñiga, Diego Guillen, Megan A. Clarke, Loretto J. Carvajal, Allan Hildesheim, John Schussler, Mónica Sierra, Teresa M. Darragh, Joel M. Palefsky, Carolina Porras, for the Costa Rica HPV Vaccine Trial (CVT) Group; Aimée R. Kreimer, Rolando Herrero

Table of contents:

Supplementary Figure 1 2

Supplementary Table 1 3

Supplementary Figure 1. Study exit algorithm applied to determine additional study visit for anal sample collection or immediate referral to high-resolution anoscopy (HRA).

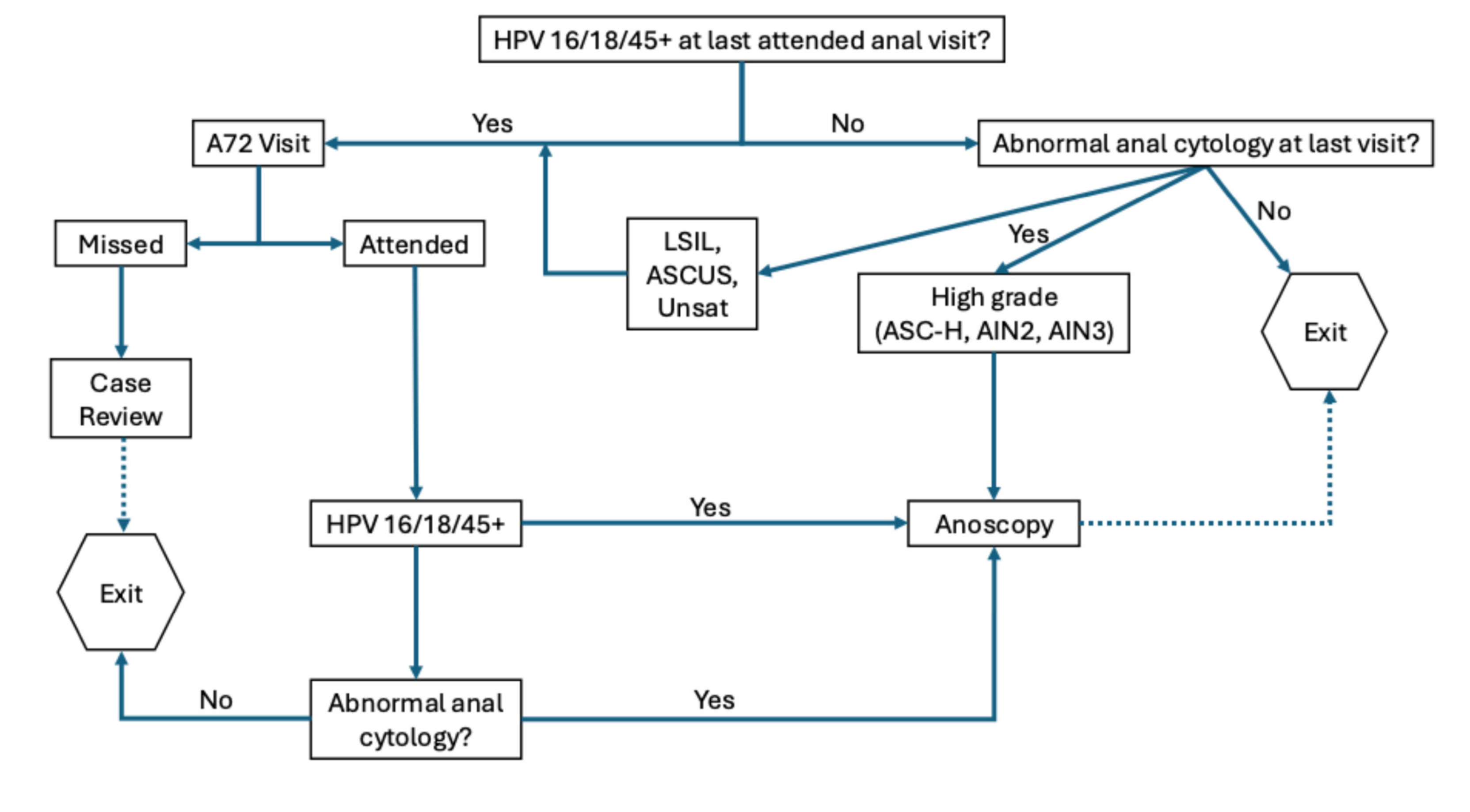

A72: Considered the fifth possible study visit with anal specimens collected, occurring between 8-10 years from original randomization.
LSIL: Low-grade squamous intraepithelial lesion
ASC-US: Atypical squamous cell of undetermined significance
Unsat: Unsatisfactory sample
ASC-H: Atypical squamous cell cannot exclude high grade
AIN2: Anal intraepithelial neoplasia, grade 2 (mild/moderate)
AIN3: Anal intraepithelial neoplasia, grade 3 (severe)
*Treatment

Supplementary Table 1. Descriptive characteristics and risk factors for meeting inclusion criteria for the anal follow-up study among women in the Costa Rica HPV Vaccine Trial based on information from the year 4 study visit.

	Univariate Prevalence Ratios (95% CI)											
	Anal HPV16+ at baseline visit		CIN3+ histology		Cervical HPV16+ at baseline visit		Anal non-16 carcinogenic HPV+ and 26+ years old at baseline		Anal low-risk HPV+ and 26+ years old at baseline		Random control (anal HPV-, 26+ years old)	
Total N	145/4,905 (3.0%)		298*/9,416 (3.2%)		404/9,404 (4.3%)		509/2,909 (17.5%)		665/2,909 (22.9%)		1,893/2,909 (65.1%)	
	n (%)	PR (95%CI)	n (%)	PR (95%CI)	n (%)	PR (95%CI)	n (%)	PR (95%CI)	n (%)	PR (95%CI)	n (%)	PR (95%CI)
Sexual history												
Lifetime number of vaginal sex partners												
Unknown	0 (0.0)	N/a	1 (0.3)	3.1 (0.4-22.8)	1 (0.2)	1.8 (0.3-13.2)	3 (0.6)	**4.3 (1.3-13.8)**	2 (0.3)	1.9 (0.5-7.9)	5 (0.3)	0.7 (0.3-1.6)
1	22 (15.2)	Ref	38 (12.8)	Ref	65 (16.1)	Ref	56 (11.0)	Ref	83 (12.5)	Ref	589 (31.1)	Ref
2	19 (13.1)	1.1 (0.6-2.0)	59 (19.8)	**2.0 (1.3-3.0)**	84 (20.8)	**1.7 (1.2-2.3)**	84 (16.5)	**1.9 (1.4-2.7)**	120 (18.0)	**1.8 (1.4-2.4)**	386 (20.4)	**0.8 (0.7-0.9)**
3+	104 (71.7)	**2.2 (1.4-3.6)**	200 (67.1)	**2.9 (2.1-4.1)**	254 (62.9)	**2.2 (1.6-2.8)**	366 (71.9)	**2.9 (2.2-3.9)**	460 (69.2)	**2.5 (2.0-3.2)**	913 (48.2)	**0.7 (0.6-0.8)**
Age at first vaginal sex	145 (100.0)	1.0 (0.9-1.0)	298 (100.0)	**0.9 (0.9-0.9)**	404 (100.0)	1.0 (1.0-1.0)	509 (100.0)	1.0 (0.9-1.0)	665 (100.0)	1.0 (0.9-1.0)	1,893 (100.0)	1.0 (1.0-1.0)
Age at first anal sex	145 (100.0)	1.0 (1.0-1.1)	298 (100.0)	1.0 (1.0-1.1)	404 (100.0)	1.0 (0.9-1.1)	509 (100.0)	1.0 (0.9-1.0)	665 (100.0)	1.0 (1.0-1.0)	1,893 (100.0)	1.0 (1.0-1.0)
Lifetime number of anal sex partners												
Unknown	2 (1.4)	1.1 (0.3-4.7)	18 (6.0)	**0.5 (0.3-0.8)**	83 (20.5)	**1.6 (1.1-2.2)**	9 (1.8)	0.9 (0.4-1.7)	10 (1.5)	0.7 (0.4-1.4)	29 (1.5)	1.1 (0.7-1.6)
0	98 (67.6)	0.8 (0.5-1.1)	221 (74.2)	1.0 (0.7-1.4)	249 (61.6)	0.8 (0.6-1.1)	326 (64.0)	**0.7 (0.6-0.8)**	442 (66.5)	**0.7 (0.6-0.8)**	1,476 (78.0)	**1.2 (1.1-1.3)**
1	32 (22.1)	Ref	43 (14.4)	Ref	59 (14.6)	Ref	130 (25.5)	Ref	169 (25.4)	Ref	333 (17.6)	Ref
2+	13 (9.0)	1.9 (1.0-3.6)	**16 (5.4)**	**1.9 (1.1-3.3)**	13 (3.2)	1.1 (0.6-2.0)	**44 (8.6)**	**1.6 (1.1-2.2)**	44 (6.6)	1.2 (0.9-1.7)	55 (2.9)	0.8 (0.6-1.0)
Any prior cervical HR-HPV+ (%)												
Negative	27 (24.1)	Ref	11 (4.1)	Ref	37 (17.7)	Ref	111 (28.3)	Ref	152 (29.0)	Ref	758 (50.9)	Ref
HPV 16	53 (47.3)	**5.9 (3.7-9.4)**	135 (50.8)	**44.6 (24.1-82.4)**	134 (64.1)	**13.1 (9.1-18.9)**	79 (20.2)	**2.2 (1.7-3.0)**	91 (17.3)	**1.9 (1.4-2.4)**	165 (11.1)	**0.7 (0.6-0.8)**
HPV18/45	9 (8.0)	1.7 (0.8-3.6)	33 (12.4)	**17.6 (8.9-34.9)**	8 (3.8)	1.3 (0.6-2.7)	52 (13.3)	**2.3 (1.7-3.2)**	69 (13.1)	**2.3 (1.7-3.0)**	103 (6.9)	**0.7 (0.5-0.8)**
Other HR-HPV	23 (20.5)	1.1 (0.6-2.0)	87 (32.7)	**12.1 (6.5-22.7)**	30 (14.4)	1.2 (0.8-2.0)	150 (38.3)	**1.8 (1.4-2.2)**	213 (40.6)	**1.8 (1.5-2.2)**	463 (31.1)	**0.8 (0.7-0.9)**
Demographics												
Marital status												
Unknown	0 (0.0)	N/a	0 (0.0)	N/a	0 (0.0)	N/a	1 (0.2)	2.2 (0.3-15.5)	1 (0.2)	1.7 (0.2-12.3)	2 (0.1)	1.0 (0.2-3.9)
Married/living with partner	78 (53.8)	Ref	192 (64.4)	Ref	232 (57.4)	Ref	322 (63.3)	Ref	406 (61.1)	Ref	1,458 (77.0)	Ref

Single	52 (35.9)	**1.8 (1.2-2.5)**	84 (28.2)	1.0 (0.7-1.2)	144 (35.6)	**1.4 (1.1-1.7)**	129 (25.3)	**1.5 (1.2-1.8)**	171 (25.7)	**1.5 (1.3-1.8)**	333 (17.6)	**0.8 (0.7-0.9)**
Divorced/separate/widowed	15 (10.3)	**1.9 (1.1-3.3)**	22 (7.4)	1.3 (0.8-2.0)	28 (6.9)	1.4 (0.9-2.0)	57 (11.2)	**1.7 (1.3-2.3)**	87 (13.1)	**2.1 (1.6-2.6)**	100 (5.3)	**0.7 (0.5-0.8)**
Smoking history												
Unknown	0 (0.0)	N/a	0 (0.0)	N/a	0 (0.0)	N/a	1 (0.2)	1.3 (0.2-9.0)	1 (0.2)	1.0 (0.1-6.8)	4 (0.2)	1.2 (0.4-3.1)
Never (%)	111 (76.6)	Ref	203 (68.1)	Ref	306 (75.7)	Ref	374 (73.5)	Ref	492 (74.0)	Ref	1,600 (84.5)	Ref
Ever (%)	34 (23.4)	1.2 (0.8-1.8)	95 (31.9)	**2.2 (1.7-2.7)**	98 (24.3)	**1.5 (1.2-1.8)**	134 (26.3)	**1.5 (1.3-1.9)**	172 (25.9)	**1.5 (1.3-1.8)**	289 (15.3)	**0.8 (0.7-0.9)**
Education												
Unknown	1 (0.7)	1.4 (0.2-10.1)	1 (0.3)	0.4 (0.1-3.0)	2 (0.5)	0.8 (0.2-3.4)	1 (0.2)	0.2 (0.0-1.6)	4 (0.6)	0.7 (0.3-1.9)	21 (1.1)	1.2 (0.8-1.9)
≤6 y	31 (21.4)	Ref	99 (33.2)	Ref	100 (24.8)	Ref	179 (35.2)	Ref	227 (34.1)	Ref	690 (36.5)	Ref
7-9 y	34 (23.4)	1.7 (1.0-2.8)	59 (19.8)	0.9 (0.7-1.3)	93 (23.0)	**1.5 (1.1-1.9)**	109 (21.4)	1.1 (0.9-1.4)	147 (22.1)	1.2 (1.0-1.5)	344 (18.2)	0.9 (0.8-1.1)
≥10 y + technical	31 (21.4)	1.2 (0.7-2.0)	66 (22.1)	0.8 (0.6-1.1)	90 (22.3)	1.1 (0.8-1.4)	105 (20.6)	1.0 (0.8-1.2)	137 (20.6)	1.0 (0.8-1.2)	422 (22.3)	1.0 (0.9-1.1)
University	48 (33.1)	**2.0 (1.2-3.1)**	73 (24.5)	0.9 (0.7-1.2)	119 (29.5)	**1.5 (1.1-1.9)**	115 (22.6)	1.0 (0.8-1.3)	150 (22.6)	1.1 (0.9-1.3)	416 (22.0)	1.0 (0.9-1.1)

Inclusion criteria are not mutually exclusive, allowing for women to be counted in multiple strata and therefore the sum of the strata is greater than the total number of women.

*28 women with CIN3+ did not have a baseline visit and were excluded from this table.

**Not assessed at L0 for UCG women.
